# *Serratia marcescens* Endocarditis with Perivalvular Abscess Presenting as Atrioventricular Block

**DOI:** 10.1155/2020/7463719

**Published:** 2020-06-11

**Authors:** Aaron Richardson, Andres Martinez, Shreya Ghetiya, Emil Missov, Robert Percy, Srinivasan Sattiraju

**Affiliations:** ^1^Division of Cardiology, University of Florida College of Medicine Jacksonville, Jacksonville, FL, USA; ^2^Department of Medicine, University of Florida College of Medicine Jacksonville, Jacksonville, FL, USA

## Abstract

*Serratia marcescens* is an aerobic, Gram-negative bacillus first identified in 1819 (Yeung et al. 2018). *S. marcescens* infective endocarditis is extremely rare accounting for only 0.14% of all cases (Phadke and Jacob 2016, Hadano et al. 2012, Nikolakopoulos et al. 2019). We present the case of a 33-year-old male with a past medical history of Hodgkin lymphoma, nonischemic cardiomyopathy ejection fraction of 25–30%, severe aortic stenosis, hepatitis C, and active intravenous (IV) drug abuse who was admitted following a motor vehicle accident. Approximately 10 days into his admission, he developed a 39.5 degree Celsius fever, which prompted collection of blood cultures. These cultures were positive (2 out of 2) for *S. marcescens* for which he was treated with intravenous cefepime. Soon after this diagnosis, patient developed a complete AV block. Given the instability of the patient, he required emergent placement of a temporary pacing wire. Transesophageal echocardiogram was ordered and revealed an aortic root abscess. Given the comorbidities and active IV drug use, conservative management was pursued. Although rare, trends suggest that this pathogen may be on the rise. Further research is needed to better understand how to effectively manage this pathogen.

## 1. Introduction

*Serratia marcescens* is an aerobic, motile, oxidase-negative, and Gram-negative bacillus first identified in 1819 [1]. *S marcescens* is an opportunistic pathogen associated with intravenous drug use, immunosuppression, previous antibiotic exposure, and indwelling catheterization. *S. marcescens* has a number of factors increasing virulence including fimbria-like adhesions, which allow for surface attachment and subsequent formation of biofilms increasing the likelihood of infection to humans. *S. marcescens* is most commonly known to cause urinary tract infections, pneumonia, and soft tissue infections. Infective endocarditis by *S. marcescens*, first described in 1951, is extremely rare accounting for only 0.14% of all cases of endocarditis [2–4].

## 2. Case Report

A 33-year-old male with a past medical history of Hodgkin lymphoma, nonischemic cardiomyopathy ejection fraction of 25–30%, severe aortic stenosis, hepatitis C, and intravenous drug abuse was admitted to the hospital following a motor vehicle accident. Upon admission, he was found to have a closed severely displaced zone III sacral fracture for which conservative management was pursued due to patient's comorbidities.

Approximately 3 days into the admission, he was diagnosed with a urinary tract infection. Broad spectrum antibiotics were ordered, and urine culture returned positive for *S. marcescens* sensitive to ciprofloxacin, gentamycin, and trimethoprim/sulfamethoxazole. Blood cultures drawn at the same time the urinary tract infection was diagnosed were negative. The patient was treated with one week of piperacillin/tazobactam for the *Serratia marcescens* urinary tract infection.

**Figure 1:**
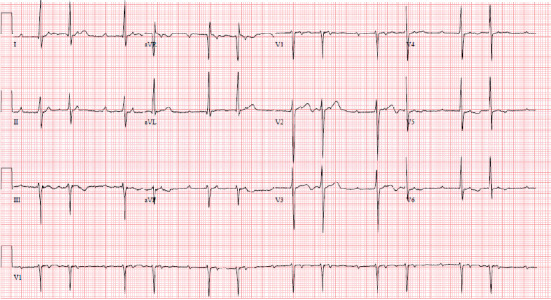
1^st^ ECG obtained from patient with a 2^nd^ degree AV block consistent with Mobitz type I.

**Figure 2:**
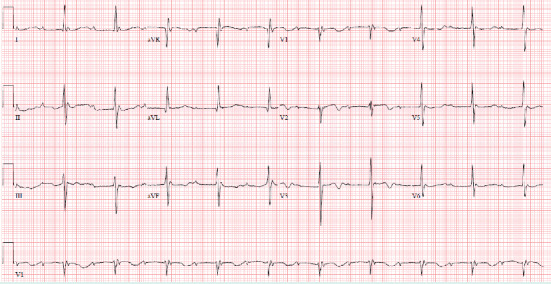
A follow-up ECG taken the next day from the patient with a 3^rd^ degree AV block.

**Figure 3:**
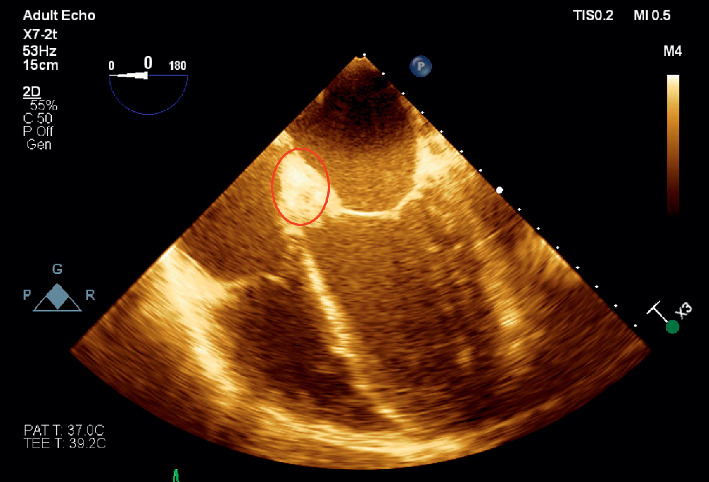
Transesophageal echocardiogram showing a 4-chamber view. Paravalvular abscess is circled.

**Figure 4:**
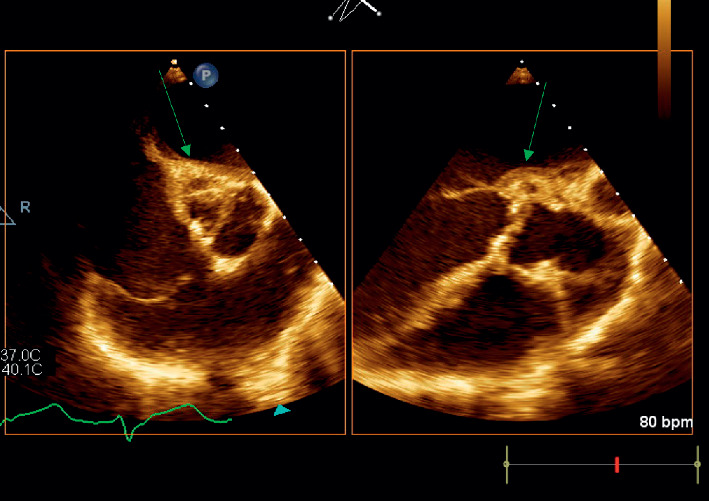
Transesophageal short axis and long axis view, respectively, of the aortic valve showing multiple echolucent areas of the aortic root suggestive of aortic root abscess.

**Figure 5:**
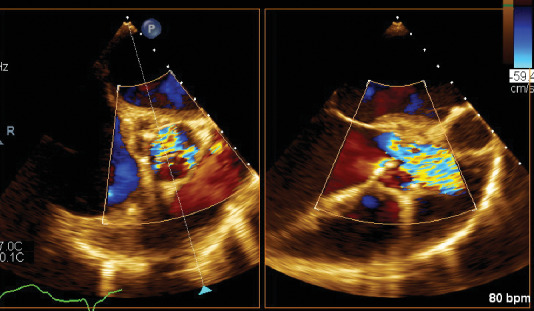
Transesophageal short axis and long axis view, respectively, of the aortic valve. Doppler interrogation shows severe aortic stenosis across valve.

**Figure 6:**
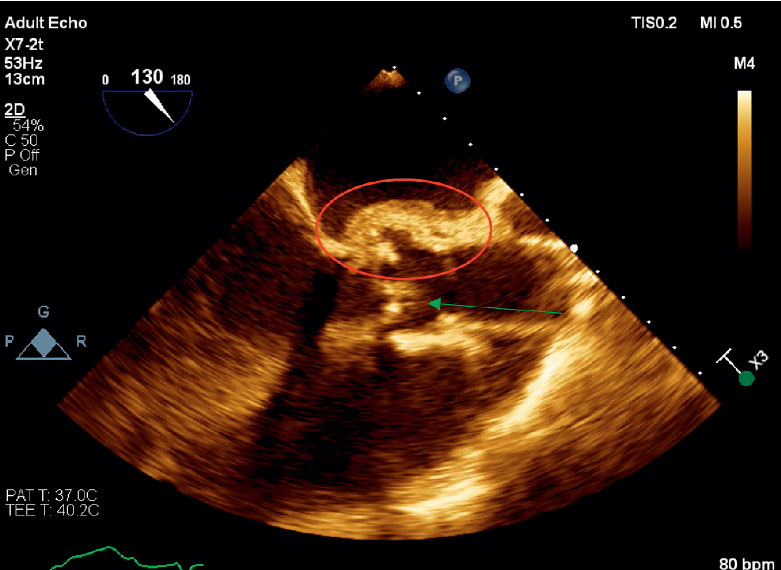
Transesophageal long axis view of the aortic valve. Paravalvular abscess is denoted by the circle. The arrow denotes vegetation on the aortic valve.

Around 10 days after the patient's diagnosis of *S. marcescens* urinary tract infection, he developed a 39.5 degree Celsius fever, which prompted collection of additional blood cultures. These cultures were positive (2 out of 2) for *S. marcescens,* same phenotype as previous urine cultures, sensitive to gentamycin, levofloxacin, and trimethoprim/sulfamethoxazole. He was treated with intravenous cefepime. One week after diagnosis of bacteremia, cardiology was consulted for bradycardia. Upon cardiology review of the initial electrocardiogram (ECG), it was found the patient had a Mobitz type 1 block (Figure 1). Patient was examined bedside and found to be asymptomatic and hemodynamically stable. A follow-up ECG was ordered the next day, which showed the patient had progressed to a complete AV block (Figure 2). Patient was hemodynamically stable when this ECG was obtained, and electrophysiology consultation was recommended. However, before the patient could be evaluated by electrophysiology, the patient had 2 symptomatic episodes of torsade de pointes. The patient was given intravenous magnesium with methadone discontinued. Given the instability of the patient, it was decided to emergently take the patient to the electrophysiology lab and place a temporary pacing wire with an external generator. Once this was completed, a transesophageal echocardiogram was ordered for evaluation of endocarditis. Transesophageal echocardiogram revealed an aortic valve area of 0.9 cm^2^ in addition to an echogenic density around the aortic root that extended into the interatrial septum. A small echolucent area was noted within this density. A small mobile echogenic density was also seen attached to the base of aortic leaflets (Figures 3–6). These findings in addition to the complete AV block and bacteremia were all consistent with an aortic root abscess. Cardiothoracic surgery was consulted; however, given patient's multiple comorbidities, conservative treatment was pursued. The patient was successfully treated with 6 weeks of intravenous cefepime in combination with 4 weeks of ciprofloxacin. Once cefepime was completed, the patient was then placed on oral antibiotic indefinitely until a possible future surgical intervention could take place.

## 3. Discussion

Endocarditis due to *S. marcescens* is indistinguishable clinically from other causes of endocarditis. Although most intravenous drug users with endocarditis have right-side involvement of the heart, *S. marcescens* has predilection for left-sided valves. *S. marcescens* endocarditis is difficult to treat due to resistance to many antibiotics including 1^st^ and 2^nd^ generation cephalosporins and penicillins. *S. marcescens* has been shown to be susceptible to 3^rd^ and 4^th^ generation cephalosporins, monobactams, carbapenems, fluoroquinolones, and aminoglycosides. Resistance to antibiotics is obtained through high-level production of chromosomal AmpC cephalosporinases combined with decreased outer membrane permeability along with the synthesis of *β*-lactamases [2, 4].

*S. marcescens* is associated with high morbidity. Paravalvular complications and valvular destruction are documented in previous cases of *S. marcescens* endocarditis. Surgical intervention is recommended within 7–10 days, especially for patients with left side involvement [1]. In fact, mortality is noted to be as high as 85% in patients with *S. marcescens* endocarditis who only received medical therapy. Treatment with only medical therapy has been more successful in those diagnosed with right-side endocarditis [3].

The 2005 Infectious Diseases Society of America (IDSA) and 2009 European Society of Cardiology make no specific recommendations on antimicrobial therapy but suggest the use of a combination of a beta-lactam and an aminoglycoside for at least 6 weeks of treatment. The most important step in treatment is to obtain cultures with sensitivities in order to appropriately focus treatment. Surgical consultation must be obtained if vegetations, paravalvular abscess, or involvement of valves are noted on echocardiogram. Failure to seek appropriate surgical treatment can result in persistent bacteremia, risk of embolization, and heart failure [1, 5].

## 4. Conclusion

*S. marcescens* endocarditis is rare, but trends suggest that this pathogen may be on the rise. This case presents the importance of early identification, culturing, and identification of antibiotic sensitivity given the resistance of this pathogen to antibiotics. No clear guidelines exist for treatment of *S. marcescens* given its rarity. Once diagnosed, early surgical consultation should be considered. Further research is needed to understand how to manage this pathogen.
